# Prediction of Chemotherapy Response in Locally Advanced Breast Cancer Patients at Pre-Treatment Using CT Textural Features and Machine Learning: Comparison of Feature Selection Methods

**DOI:** 10.3390/tomography11030033

**Published:** 2025-03-13

**Authors:** Amir Moslemi, Laurentius Oscar Osapoetra, Archya Dasgupta, Schontal Halstead, David Alberico, Maureen Trudeau, Sonal Gandhi, Andrea Eisen, Frances Wright, Nicole Look-Hong, Belinda Curpen, Michael Kolios, Gregory J. Czarnota

**Affiliations:** 1 Physical Sciences, Sunnybrook Research Institute, Toronto, ON M4N 3M5, Canada; amir.moslemi@sunnybrook.ca (A.M.); laurentiusoscar.osapoetra@sunnybrook.ca (L.O.O.); archya1010@gmail.com (A.D.); schontal.halstead@gmail.com (S.H.); david.alberico@sri.utoronto.ca (D.A.); 2 Department of Medical Oncology, Department of Medicine, Sunnybrook Health Sciences Centre, Toronto, ON M4N 3M5, Canada; maureen.trudeau@sunnybrook.ca (M.T.); sonal.gandhi@sunnybrook.ca (S.G.); andrea.eisen@sunnybrook.ca (A.E.); 3 Department of Medicine, University of Toronto, Toronto, ON M4N 3M5, Canada; 4 Department of Surgical Oncology, Department of Surgery, Sunnybrook Health Sciences Centre, Toronto, ON M4N 3M5, Canada; frances.wright@sunnybrook.ca (F.W.); nicole.lookhong@sunnybrook.ca (N.L.-H.); 5 Department of Surgery, University of Toronto, Toronto, ON M4N 3M5, Canada; 6 Department of Medical Imaging, Sunnybrook Health Sciences Centre, Toronto, ON M4N 3M5, Canada; belinda.curpen@sunnybrook.ca; 7 Department of Medical Imaging, University of Toronto, Toronto, ON M4N 3M5, Canada; 8 Department of Physics, Toronto Metropolitan University, Toronto, ON M5B 2K3, Canada; mkolios@torontomu.ca; 9 Department of Radiation Oncology, Sunnybrook Health Sciences Centre, Toronto, ON M4N 3M5, Canada; 10 Department of Radiation Oncology, University of Toronto, Toronto, ON M4N 3M5, Canada; 11 Department of Medical Biophysics, University of Toronto, Toronto, ON M4N 3M5, Canada

**Keywords:** NAC, LABC, CT imaging, textural features, machine learning

## Abstract

Rationale: Neoadjuvant chemotherapy (NAC) is a key element of treatment for locally advanced breast cancer (LABC). Predicting the response of NAC for patients with LABC before initiating treatment would be valuable to customize therapies and ensure the delivery of effective care. Objective: Our objective was to develop predictive measures of tumor response to NAC prior to starting for LABC using machine learning and textural computed tomography (CT) features in different level of frequencies. Materials and Methods: A total of 851 textural biomarkers were determined from CT images and their wavelet coefficients for 117 patients with LABC to evaluate the response to NAC. A machine learning pipeline was designed to classify response to NAC treatment for patients with LABC. For training predictive models, three models including all features (wavelet and original image features), only wavelet and only original-image features were considered. We determined features from CT images in different level of frequencies using wavelet transform. Additionally, we conducted a comparison of feature selection methods including mRMR, Relief, Rref QR decomposition, nonnegative matrix factorization and perturbation theory feature selection techniques. Results: Of the 117 patients with LABC evaluated, 82 (70%) had clinical–pathological response to chemotherapy and 35 (30%) had no response to chemotherapy. The best performance for hold-out data splitting was obtained using the KNN classifier using the Top-5 features, which were obtained by mRMR, for all features (accuracy = 77%, specificity = 80%, sensitivity = 56%, and balanced-accuracy = 68%). Likewise, the best performance for leave-one-out data splitting could be obtained by the KNN classifier using the Top-5 features, which was obtained by mRMR, for all features (accuracy = 75%, specificity = 76%, sensitivity = 62%, and balanced-accuracy = 72%). Conclusions: The combination of original textural features and wavelet features results in a greater predictive accuracy of NAC response for LABC patients. This predictive model can be utilized to predict treatment outcomes prior to starting, and clinicians can use it as a recommender system to modify treatment.

## 1. Introduction

Locally advanced breast cancer (LABC) is a heterogeneous disease with a wide variety of clinical presentations [1,2]. LABC refers to locally advanced breast cancer, which includes any tumor that is larger than 5 cm or that involves the skin or the chest wall [1,2]. LABC also encompasses inflammatory breast cancer and cases where patients have fixed axillary lymph nodes or involvement of the ipsilateral supraclavicular, infraclavicular, or internal mammary lymph nodes [1,2]. LABC tumors present a significant clinical challenge, as patients with locally advanced disease generally have lower survival rates compared to those with early-stage breast cancer [1,2]. The standard treatment for LABC includes a multimodality treatment comprised of systemic therapy, surgery, and radiotherapy [1,2]. The resection of inoperable tumors in selected patients is feasible as neoadjuvant chemotherapy (NAC) enhances tumor regression. This is subsequently followed by surgery, adjuvant radiotherapy, and, if appropriate, targeted therapy or endocrine therapy [3].

LABC tumors treated with NAC present variable responses, with only 15–40% of patients finally attaining pathological complete response to therapy [4]. Tumor pathological response to NAC has been shown as an essential prognostic indicator for long-term disease-free survival (DFS) and overall survival (OS) in a specific group of patients [5,6]. However, the treatment response evaluation of LABC tumors to NAC is typically performed at the end of the treatment period, several months following the commencement of treatment. The assessment relies on pathological evaluations—typically, a Miller–Payne (MP) grading system is employed to evaluate tumor cellularity by comparing pre-treatment core-needle biopsies with post-treatment surgical specimens [6,7]. However, because of the invasive nature of these approaches, non-invasive imaging techniques to assess treatment responses in LABC tumors are sought. Imaging features that can predict tumor responses at early stages of NAC could steer personalized treatments.

Histopathological analysis and quantitative imaging techniques have provided insights into various characteristics used to assess the response of LABC tumors to NAC, particularly by applying artificial intelligence [8,9]. LABC tumors responsive to NAC exhibited less cell proliferation in contrast to those of non-responders, attributed to the increase in apoptosis [10,11]. In addition, a study showed a correlation between human epidermal growth factor receptor 2 (HER2) expression and response to NAC [12]. HER2-positive tumors demonstrate considerably higher rates of attaining pathological complete response than those of HER2-normal tumors [12]. Previous studies using diffuse optical spectroscopic techniques reported a significant difference in changes of hemoglobin contents after 1 week of therapy between complete pathological response in contrast to those with partial pathological response [13,14,15]. Other studies that employed magnetic resonance imaging (MRI) [16] and circulating DNA and RNA-integrity quantifications [17] assessed response prediction after the commencement of chemotherapy.

According to the St-gallen Guidelines [18], the LABC subtypes were categorized into four molecular subtypes, including Luminal A (ER+, PR+, HER2- and Ki-67 < 14%), Luminal B (ER+, HER2+, any Ki-67 and any PR), HER2-enriched (ER-, PR- and HER2+), and triple negative (ER-, PR- and HER2-).

Recently, radiomics has emerged as a promising field in quantitative imaging [19,20,21] Radiomics aims to enhance the existing imaging data through the automatic determination of a large number of features using advanced feature analysis techniques [19,20,21]. Previous studies have elucidated the potentials of radiomics for LABC treatment outcome prediction using different imaging modalities [22,23].

Therapy response to breast cancer analysis using imaging techniques has been investigated in several studies based on tumor size variation [24]. Imaging approaches such as dynamic contract-enhanced magnetic resonance imaging (DCE-MRI) [16], positron emission tomography (PET) [25,26], diffuse optical imaging (DOI) [26,27], ultrasound (US) imaging [28,29] and quantitative ultrasound [30,31,32,33] have been utilized as imaging techniques to evaluate treatment response to breast cancer. Although effectiveness and promising results are reported in all these studies, the price and accessibility of DCE-MRI, price and radionuclide injection of PET, resolution of volumetric DOI images and quality of 3D volumetric US images are the main challenges for these imaging techniques.

Quantitative imaging has attracted attention to assess the response to chemotherapy in patients with LABC. The reported results of QUS and DOI are promising for predicting therapy response prior to the initiation of treatment [22,23]. The findings from these studies indicated a tumor aggressiveness and responsiveness to chemotherapy and the micro-structure and metabolic characteristics of tumors. Quantified features are determined from QUS and DOI images and used to predict the treatment response.

Computed tomography (CT) is a powerful imaging technique used to provide 3D volumetric images to analyze the micro-characteristics of a cancerous tumor. Quantitative CT (qCT) is employed to quantify CT images to extract informative features for treatment response prediction. Although the resolution of CT is considerably larger than cellular dimensions, the correlation between CT voxel intensities and tissue micro-structure can be used to interpret tumor response. In keeping with this, qCT has been used for disease diagnosis [34], disease discrimination [35] and disease progression prediction [36]. In the context of treatment response analysis, contrast-enhanced CT (CE-CT) is used to analyze cancer response to NAC for breast cancer [37], and textural qCT is used to predict response to NAC treatment for LABC patients prior to the start of treatment [38]. Zhange et al. [39] applied ResNet34 to extract deep radiomics features from DCE-MRI to predict axillary response after NAC. Yongfeng et al. [40] extracted radiomics features from MRI and trained multivariate logistic regressions to predict early response to NAC treatment. Yu et al. [41] used deep learning radiomics to extract deep features from pretreatment ultrasound images using deep convolutional neural networks to evaluate NAC response. Oda et al. [42] extracted CT radiomics features to build a machine learning model to predict the pathological response of NAC. Identifying an optimized predictive model that incorporates comprehensive and discriminative features stands as the primary void in research on predicting NAC outcomes. Selecting the most important and discriminative features significantly affects the training phase of machine learning in terms of the overfitting challenge. Although machine and deep learning have received great attention for predicting treatment outcomes, overcoming overfitting and achieving effective generalization remain persistent problems.

In this study, to address the above limitation, we proposed a machine learning model to predict outcomes of NAC for patients with LABC based on determined radiomics features of CT at different levels of frequencies. Therefore, we hypothesize that CT texture features of LABC tumors provide prognostic indications for assessing therapy response. We utilized the pool of radiomics features for building a multi-variate classification models to predict response. This study builds upon a preliminary study on quantitative CT (qCT) for LABC response prediction reported earlier [38]. Here, we expanded the cohort size, determined more radiomics features (specifically, the inclusion of wavelet features), and improved model building and evaluation strategies. Specifics on the improvements include the following: data preprocessing and the evaluation of different feature selection methods. We applied the most recent advanced feature selection methods to improve the performance of the classifier. To show the effectiveness of wavelet features, we considered three models to build the classifier, including only original CT image features, only wavelet features and a combination of original CT image features and wavelet features.

## 2. Material and Methods

### 2.1. Study Protocol and Data Acquisition

This research was carried out in compliance with the guidelines established by the institution ethics guidelines at Sunnybrook Health Sciences Center (SHSC). This study enrolled a total of 117 patients (82 responders and 35 non-responders) with LABC undergoing NAC (in the timeline 2019–2022). All experimental protocols were approved by SHSC, and consent was obtained from all subjects. The inclusion of patients in this study was contingent upon obtaining written informed consent. Tumor sizes were obtained from magnetic resonance imaging (MRI) scans performed as part of patients’ standard of care. Histopathological analysis of pre-treatment core-needle biopsy specimens confirmed the cancer diagnosis for all patients. The specimens provided information regarding the primary cellularity, tumor subtype, and hormone receptor status expressions that include estrogen receptor (ER), progesterone receptor (PR), and HER2 expressions. All patients completed a full course of NAC that lasted commonly for 4–6 months. Subsequently, these patients underwent either lumpectomy or mastectomy. After surgery, adjuvant therapies that consisted of radiation, maintenance Transtuzumab for HER2-positive tumors or endocrine therapy (for hormonal-receptor-positive tumors) were initiated as per standard institutional practice. The oncology treatments consist of AC-T or FEC-D chemotherapy +/− Herceptin, which continue to be administered to a majority of chemotherapy-naive patients with locally advanced breast cancer. This is carried out at Sunnybrook Health Sciences Centre based on guidelines from Cancer Care Ontario. This study did not include smaller tumors on purpose since those are handled differently. If primary tumor sizes were small, they were accompanied by un-reselectable bulky lymph nodes to meet the criteria of locally advanced breast cancer.

Based on institutional standard of care, pre-treatment CE-CT images of the breast were obtained for all patients with LABC. The multi-slice CT scanner (LightSpeed, GE Medical Systems, Chicago, IL, USA) had the following scan parameters: tube voltage—120 kV, X-ray tube current—10–367 mA, slice thickness—2.5 mm, pixel spacing—0.8 × 0.8 mm, and slice size—512 × 512 pixels, which were applied in helical mode. To measure tumor size and evaluate chest wall involvement, patients underwent clinical MRI scans before and after treatment, following the institutional standard of care for patients with LABC.

### 2.2. Pathological Evaluation of Tumor Response

The patients received either lumpectomy or mastectomy after completing a full course of NAC. Standard histopathologic procedures were employed to evaluate for tumor pathological response to NAC as part of clinical care. Patients were classified into two groups—non-responders (‘NR’) and responders (‘R’)—using a modified response (MR) grading system based on the Response Evaluation Criteria in Solid Tumor (RECIST) [24] and the residual tumor cellularity [6]. RECIST assesses the percent change in tumor size (in its longest dimension) from pre-treatment and post-treatment time points.

An MR score of 1 is associated with no reduction in tumor size. An MR score of 2 was associated with a reduction in tumor size up to 30%. An MR score of 3 was associated with a reduction in tumor size between 30 and 90%. An MR score of 4 was associated with a reduction in tumor size of more than 90%. Lastly, an MR score of 5 was associated with no evidence of residual tumor at all.

Alongside these RECIST-based criteria, the residual tumor cellularity was also taken into account in order to assess response. Here, a threshold of 5% for tumor cellularity was utilized. Responder tumors are those with residual cellularity less than or equal to 5% (<=5%); otherwise, tumors were non-responders based on only cellularity criterion. The overall response combined both RECIST-based criteria related to tumor size reduction and residual tumor cellularity. A RECIST criterion considers a patient as a responder (‘R’) if either the reduction in tumor size was greater than 30% (MR score 3–5) or residual tumor cellularity was low (<=5%). A patient was a non-responder (‘NR’) if the reduction in tumor size was below 30%, or there was an enlargement in tumor size (MR score 1–2). We used both RECIST-based criteria and residual tumor cellularity to establish the target response for binary classification.

### 2.3. Feature Determination and Pre-Processing

The regions of interest (ROIs) were manually specified for all CT image slices to cover the whole tumor. Trained staff under the supervision of expert oncologists performed all the tumor segmentations in 3D CT images (a tumor of each slice is segmented). Texture-based CT radiomic features were determined using a Pyradiomics Python package [43]. Radiomics features were determined for both images and wavelet-based decomposed images to obtain comprehensive information about the images. The determined texture-based CT radiomics features were 14 shape-based features, 19 first-order statistics features, 24 gray-level co-occurrence matrix (GLCM) features [44], 16 gray-level run-length matrix (GLRLM) features [45], 16 gray-level size-zone matrix (GLSZM) features [44], 14 gray-level dependent matrix (GLDM) features [44] and 5 neighboring gray-level dependence matrix (NGLDM) features [46]. All features were determined for image and wavelet decompositions, but shape-based features were not considered for wavelet decompositions. Table 1 shows the determined features from each texture-based CT radiomic.

### 2.4. Feature Determination Using Wavelet Transform

Feature determination was carried out at different levels of spatial frequency using wavelet transform. Shift variance is the main drawback of discrete wavelet transform (DWT), which should be suppressed. Aiming to tackle this defect, stationary wavelet transform (SWT), which is the translation-in-variance modification of DWT, was applied to decompose images to different level of frequencies. Therefore, the pyradiomics-PyWavelets Python package was applied to extract radiomics features at different levels of frequencies [43]. Then, images were decomposed to eight coefficients (LLL, LLH, LHL, LHH, HLH, HLL, HHL and HHH) using two-level wavelet decomposition. In this study, the ‘haar’ mother wavelet function and two-level decomposition were considered for radiomics feature determinations. Details of the wavelet transform for feature determination are provided in the Supplementary Materials (Section S1 and Figure S1).

Therefore, 107 and 93 features were determined for original images and wavelet decompositions, respectively.

### 2.5. Feature Selection

All feature selection and standardization were performed on the training set. All features were normalized using Z-score standardization (mean subtraction and normalization to the standard deviation for each feature). Data were randomly divided into training and test sets in order to reduce the chance of bias in the training set, and the mean and maximum value of performance were reported. Feature selection is categorized as one of the most important steps of data preprocessing in order to decrease the dimension of data, reduce the probability of overfitting and obtain the most discriminative features.

#### Feature Selection Techniques

Feature selection techniques follow the three strategies, including filter, wrapper and embedded. In the filter strategy, features are selected independent of the classifier, such as Laplacian score feature selection. In the wrapper strategy, features are selected based on the performance classifier, such as sequential feature selection [47]. In the embedded strategy, feature selection is a part of the training process, such as a decision tree. In terms of label information, feature selection is classified into supervised, unsupervised and semi-supervised [47]. In this study, we applied five different feature selection techniques.

minimal-Redundancy-Maximal-Relevance (mRMR) [48]: mRMR is a filter-based supervised feature selection technique. mRMR ranks the features by maximizing the mutual information between features and the labels while minimizing mutual information among the selected features themselves.

Relief [49]: Relief is a filter-based supervised feature selection technique. Relief obtains the best features based on their ability to distinguish between instances.

Perturbation-based feature selection (PFS) [50]: PFS is a filter-based supervised feature selection technique. PFS obtains uncorrelated features based on perturbation theory by solving the least-square problem for data and perturbed data (the matlab code can be found at http://github.com/majid1292/DRPT (accessed on 28 February 2025).

Reduced row echelon form (Rref) [51]: Rref is a filter-based supervised feature selection technique. Rref sorts all features based on information gain and applies reduced row echelon form to extract all independent features.

QR feature selection (QR) [52]: QR is a filter-based unsupervised feature selection technique. QR works based on matrix factorization. In this technique, data are decomposed to column space and null space, such that all information is embedded in column space. The features corresponding to column space are obtained by a permutation matrix.

Nonnegative matrix factorization feature selection (NMFFS) [53]: NMFFS is a filter-based unsupervised feature selection technique. NMFFS decomposes data to a feature weight matrix and representation matrix, which are nonnegative. Features are ranked by considering orthogonality constraints on the feature weight matrix (the pseudo code can be found in Algorithm 1 [53]).

(Details of each feature selection method can be found in Supplementary Materials S2).

### 2.6. Training Model

Due to the imbalance in our data, the classifier’s performance is significantly impacted. In order to tackle this challenge in the training phase, we employed the SMOTE method for the oversampling of the minority group [54]. This up-sampling in the training phase improves the learning process for classifiers to discriminate responders and non-responders. After ranking features, Top-5, Top-10 and Top-15 features were considered to train the classifier.

### 2.7. Response Prediction

The three classifiers included K-nearest neighbor (KNN), support vector machine (SVM) with RBF kernel, and decision tree (DT), which were employed for classifying patients with a response and those without. All the hyperparameters of KNN, SVM and DT are tuned using a grid search. SVM with RBF kernel has two hyperparameters (‘C’, the trade-off between non-separable samples and the complexity of the algorithm, and ‘gamma’, which is the radius of the RBF kernel) that were tuned by grid search. Hyperparameter tuning significantly affects the performance of classifiers. SVM works based on risk minimization by finding a hyperplane to discriminate between responders and non-responders, and it is robust against overfitting.

Three models were developed, including image features, wavelet features, and the combination of image and wavelet features. Additionally, two data splitting strategies were compared, including hold-out splitting (75% train and 25% test) and leave-one-patient-out (LOPO) splitting. For hold-out splitting, 50 times runs were done and mean and maximum values were reported.

### 2.8. Evaluation Metric

Accuracy, sensitivity, specificity, and balanced-accuracy were used to evaluate the performance of classifiers on the test data, expressed as follows.

$$Accuracy=\frac{TP+TN}{TP+TN+FP+FN}$$

$$Sensitivity=\frac{TP}{TP+FN}$$

$$Specificity=\frac{TN}{TN+FP}$$

$$Precision=\frac{TP}{TP+FP}$$

$$Blanced-Accuracy=\frac{Sensitivity+Specificity}{2}$$

where *TP*, *TN*, *FP* and *FN* indicate true positive (true response), true negative (true non-response), false positive and false negative, respectively.

**Figure 1:**
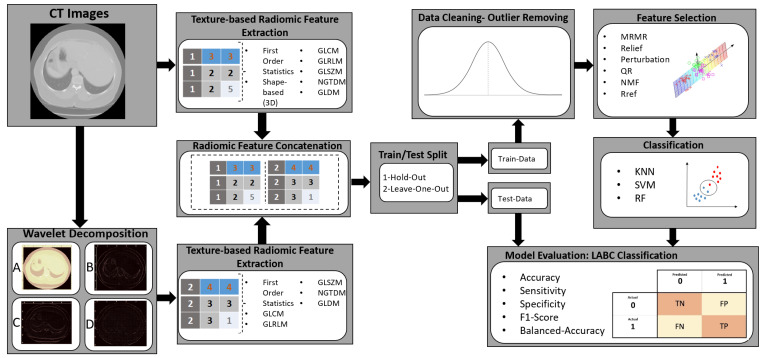
This figure shows the method to extract features, concatenating features from original image and wavelet decomposition, and training machine learning with these features to predict treatment response. In wavelet decomposition, coefficient (**A**) represents approximation of image, coefficient (**B**) represents horizontal detail, coefficient (**C**) represents vertical of image and coefficient (**D**) represents diagonal detail of image.

The schematic of the proposed method to classify the patients with a response and those without a response is shown in Figure 1.

## 3. Statistical Analysis

All statistical analysis was performed using the MATLAB 2019 Statistics and Machine Learning Toolbox™ (ver. 9.6.0.1072779 R2020b, The MathWorks, Inc., Natick, MA, USA). An unpaired *t*-test was applied to statistically compare the selected features in the two response cohorts.

## 4. Implementation of Method

Feature determination was implemented in Python using PyRadiomics version 3.0.1. The feature selection and classification were implemented using MATLAB R2020b (MathWorks Inc., MA, USA). The codes were implemented using Intel(R) Core (TM) i7-1065G7 CPU 1.5 GHz CPU and 16 GB Ram.

## 5. Results

The participants of this study included 117 women with a mean age of 52 ± 11 (standard deviation) years. The majority of participants (n = 82) had a clinic–pathological treatment response (partial or complete response), in contrast to 35 women who had no treatment response (stable disease or progressive disease), as defined by RECIST criteria [24]. Invasive ductal carcinoma (IDC) was the major histopathology for patients, and a minority of the patients were diagnosed with invasive lobular carcinoma (ILC) and invasive metaplastic carcinoma (IMC). A majority of patients had positive estrogen (ER+) and progesterone (PR+) receptors in tumors, which were found to be major molecular features in patients, and positive Her2/Neu (HER2+) receptors and triple-negative tumors (ER-, PR-, HER2-) were found in a minority of patients. On average, the tumor sizes changed from 5.2 cm to 1.4 cm for responders and from 5.6 cm to 6 cm for non-responders. The chemotherapy regimens used were doxorubicin (Adriamycin); cyclophosphamide followed by paclitaxel (Taxol) (AC-T); 5-fluorouracil, epirubicin, and cyclophosphamide followed by docetaxel (FEC-D); doxorubicin and cyclophosphamide followed by docetaxel (Taxotere) (AC-D); and paclitaxel and cyclophosphamide (TC). Furthermore, the monoclonal antibody tratuzumab (Herceptin) (TRA) was also used for LABC patients with HER2+ tumors. There was no therapy modification based on imaging during this observational study. The pathological and clinical characteristics of patients are summarized in Table 2. We applied a *t*-test between two groups, which is shown in Table 2 (statistically significant results are denoted by †).

In total, 851 radiomic biomarkers were determined from CT images and wavelet coefficients. Table 3 presents the classification scores of the classifiers for predicting treatment response using hold-out data splitting for three models. The mean (after running 50 times) and the maximum specificity, sensitivity, accuracy and balanced-accuracy are reported in Table 3 (details provided in Supplementary Materials Figures S2 and S3, and Table S1).

Table 4 shows the LOPO splitting performance (details provided in Supplementary Materials Tables S2–S4).

In terms of response prediction for LOPO data splitting, using the Top-5 features of model 3 (image and wavelet features), which are ranked by mRMR, and classifying by KNN, the best results were achieved compared to other techniques (accuracy = 75%, specificity = 76%, sensitivity = 62%, and balanced-accuracy = 72%). For hold-out splitting, using the Top-5 features of model 3 (image and wavelet features), ranked by mRMR, and classifying by KNN achieved the best result compared to other techniques (accuracy = 77%, specificity = 80%, sensitivity = 56%, and balanced-accuracy = 68%). Results are the mean of 50 runs using data randomly split to the training and test sets. Based on the sensitivity metric, the model was robust against imbalanced challenge. Both the SMOTE technique and effective feature selection contributed to the robustness of the model. The balanced accuracy shows the capability of model to predict minor classes and major classes. Based on balanced accuracy, our predictive model is robust to predict both the minor class and major class.

**Figure 2:**
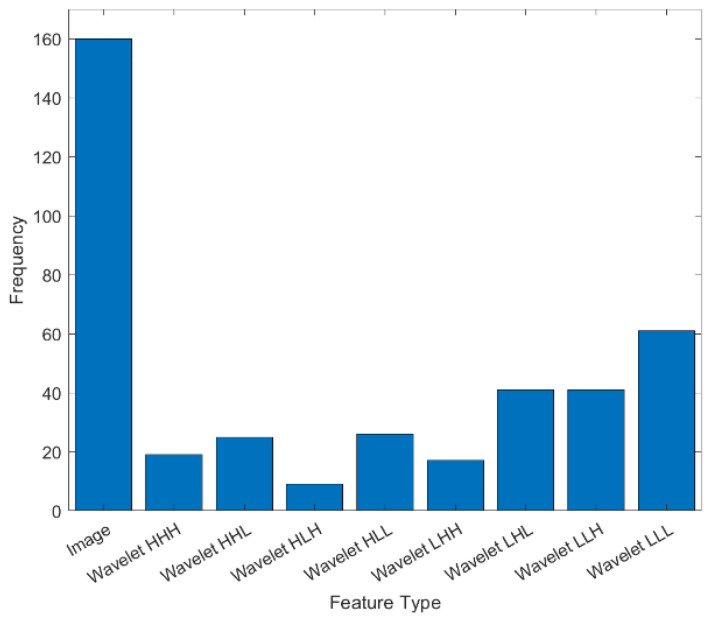
This figure illustrates the frequency of selected features for 50 times runs. It shows the frequency of selected features for extracted radiomic features from original image and each wavelet coefficients (LLH, LHL, LHH, HLL, LLH, HHL, HHH, LLL).

**Figure 3:**
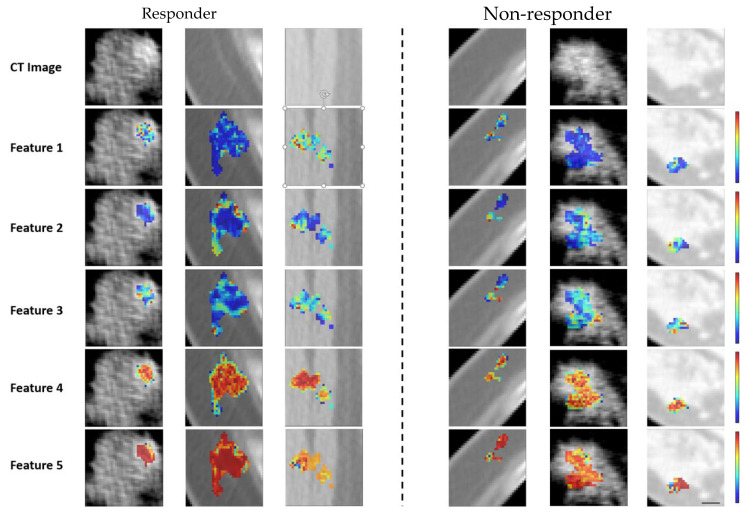
Parametric maps for the two response groups: Representative CT images and parametric overlaid map for a responding and a non-responding patient. The parametric maps demonstrate first order Kurtosis original image (Feature 1 with range [0–4]), GLRLM grey level variance of original image (Feature 2 with range [0–35]), first order robust mean absolute deviation HLL (Feature 3 with range [0–18]), Wavelet-LLH-GLDM- Dependence Entropy (Feature 4 with range [0–4]) and GLCM cluster shade LLL (Feature 5 with range [−14,000–0]).

The histogram of selected features is shown in Figure 2, which presents the frequency of selected features using mRMR. The frequency of selected features for images and coefficients of wavelets is separately provided in the Supplementary Materials (Figures S2–S5). They show the five most-ordered Kurtosis original image, the GLRLM grey-level variance of the original image, first-order robust mean absolute deviation HLL, wavelet-LLH-GLDM-dependence entropy and GLCM cluster shade LLL. Figure 3 presents representative CT images with parametric feature maps overlaid, generated using the Top-5 selected features.

A two-sided *t*-test (statistical test) was applied in order to assess the top selected radiomics biomarkers in the responder patients and non-responder patients. Results of this statistical test showed that the wavelet-LLH-GLDM-dependence entropy feature was the statistically significant feature, with a *p*-value of 0.04. The *p*-value for all top features is provided in the supplementary material (Supplementary Materials Table S5). Other features on their own were not statistically significant but needed to be used in combination for the classification of patients to occur. Three wavelet-based radiomics features were found among the Top-5 selected features, which shows the important role of wavelet features in improving the machine learning classifier.

### Feature Selection Techniques Comparison

Table 5 presents a comparison of six different feature selection methods. The top results were achieved using a combination of original features and wavelet features. Among the methods, mRMR ranked first with an accuracy of 75%, specificity of 76%, sensitivity of 62%, and balanced accuracy of 72%. QR ranked second with an accuracy of 82%, specificity of 39%, sensitivity of 70%, and balanced accuracy of 60%. NMFFS ranked last, with an accuracy of 71%, specificity of 35%, sensitivity of 60%, and balanced accuracy of 54%.

## 6. Discussion and Conclusions

The results found in this research indicate for the first time that CT-based textural features at different frequency levels can be applied to predict LABC responses to NAC before the start of treatment. In this study, CT images of 117 patients with LABC were collected before chemotherapy initiation. Response assessments were conducted after neoadjuvant chemotherapy treatment using a standard clinical methodology. Specifically, the response of chemotherapy treatment was specified after the completion of the course of NAC based on standard clinical (RECIST) and histopathological methods. Radiomic features were determined using first-order statistics, shape, GLCM, GLRLM, GLSZM, GLDM and NGLDM from CT images and wavelet decompositions of CT images. In total, 851 features were obtained, and four filter-based feature selections (including mRMR, perturbation-based techniques, relief and QR) were used in order to rank the features. The three classifiers included decision tree (DT), support vector machine (SVM) and k-nearest neighbor (KNN) methods, which were applied to predict the response of treatment. The best performance for hold-out splitting was obtained by the mRMR-KNN methodology using Top-5 features, with a mean accuracy and balanced accuracy of 77 and 68%, respectively. The best performance for LOPO splitting was obtained by the mRMR-KNN methodology using Top-5 features, with a mean accuracy and balanced accuracy of 75% and 72%, respectively.

The details of cellular structures cannot be visualized using clinical CT due to spatial resolution limitations. Previous studies demonstrated that there is a correlation between cellular micro-structure characteristics and tumor response [23,55,56,57]. However, the voxel intensity of CT images, which carries information linked to tissue attenuation coefficients, can be used to detect variations in tissue micro-structure [58]. Therefore, tumor tissue micro-structure can be characterized using textural features quantification at the CT resolution limit. Textural features are able to quantify CT voxel intensities to determine spatial variations information which can be used to analyze tumor structure and link it to response through correlation. Therefore, quantitative CT biomarkers can be leveraged to discriminate responder LABC patients form non-responder. The results of this study indicate that features from wavelet decompositions are sufficient to distinguish responder patients from non-responder patients predictively ahead of their chemotherapy. Specifically, the wavelet-LLH-GLDM-dependence entropy feature was the only feature with a *p*-value less than 0.005.

Feature selection significantly influences the performance of learning algorithms. Redundant and multicollinear features increase the probability of overfitting for learning algorithms. Therefore, the most informative features are identified by feature selection. The results in this work demonstrate that mRMR achieved the best performance compared with other feature selection techniques. mRMR obtains informative features by minimizing the redundancy amongst features and maximizing the relevancy between features linked to target conditions simultaneously. The sensitivity to multicollinearity for classifiers is different. DT is robust to multicollinearity since, as an algorithm, it is categorized as an embedding learning algorithm. In DT, feature selection is a main part of classification, such that features for splitting are chosen based on carried information. However, KNN and SVM can be highly sensitive to multicollinearity since there is no feature selection or feature ranking as part of learning.

Comparison among feature selection techniques shows the effectiveness of mRMR. However, NMFFS was not effective approach. NMFFS proposed that the orthogonality constraint could transform the feature weight matrix into an indicator matrix, making it suitable for feature selection. However, Saberi et al. [59] demonstrated that the orthogonality constraint alone is insufficient to generate an indicator matrix.

Classes in this study were not balanced, and the number of responding patients was the major label. Aiming to tackle this challenge, a SMOTE technique was used to oversample the minority class [60,61]. This technique decreases overfitting due to a majority group and improves classification accuracy.

The effectiveness of imaging and textural analysis to predict chemotherapy treatment outcomes has been researched in several studies. Sadeghi et al. [27] determined textural features using GLCM methods from diffuse optical spectroscopic (DOS) images to predict NAC response for 12 patients with LABC. They showed that DOI-based textural and mean-value parameters can differentiate responder and non-responder patients. Tran et al. [22] applied different classifiers, including logistic regression, naive Bayes type, and KNN, to classify 37 LABC patient in terms of responding to NAC treatment using GLCM textural features determined from DOS. Tadayyon et al. [23] determined texture features from quantitative ultrasound (QUS) to predict NAC for 56 LABC patients. They showed that QUS texture and image quality features can be effective predictors of tumor response to NAC. Dastjerdi et al. [62] leveraged quantitative CT (qCT) to predict the response of NAC for 72 LABC patients by machine learning and demonstrated the effectiveness of qCT for response prediction. They determined GLCM features for all 2D CT slices and used mRMR to rank features and SVM, DT, multilayer perceptron (MLP), and random forest as classifiers to discriminate responders from non-responders. Likewise, Dastjerdi et al., in another study, applied second-order GLCM to predict the NAC response for LABC patients [57]. In second-order GLCM, GLCM is applied on a GLCM parametric map for a second time. Teruel et al. [63] determined 16 GLCM textural features from dynamic contrast-enhanced MRI (DCE-MRI) images to predict the pathological responses of 58 LABC patients to NAC treatment. They found eight features that are statistically significant in distinguishing responder patients from non-responders. Cheng et al. [64] designed a study using 61 patients to predict the pathological complete response (pCR) to NAC using ^18^F-FDG PET/CT and textural features. They determined the maximum standardized uptake value, metabolic tumor volume and total lesion glycolysis as imaging parameters and entropy, coarseness and skewness as textural features to analyze the pCR. Consequently, the results indicated that variations in textural features after two cycles of treatment could be found in both HER2- and HER2+ patients. The combination of radiomics features and machine learning improved the efficiency of outcome prediction for breast cancer [65].

Nevertheless, a small population can be main challenge for all these studies since the generalization of machine learning algorithms can be directly affected by population size. Secondly, these studies only consider GLCM as textural features, whereas in the past, investigators have considered GLSZM, GLRLM, NGTDM and GLDM in addition to GLCM. GLSZM, GLRLM, NGTDM and GLDM provide information about the size of adjacent pixels, the length of consecutive pixels, the disparity between a particular gray level intensity value, the average intensity value of neighboring pixels and the relative frequency of gray level intensity, respectively. Furthermore, this work determined all of these textural features at different frequencies using wavelet decomposition to provide comprehensive information. Last but not least, this work determined 3D textural features, in contrast to other studies that determined 2D textural features [57,62]. Three-dimensional textural features provide 3D volumetric region-of-interest (ROI) information, which is more comprehensive than 2D. The superiority of 3D radiomics features over 2D radiomics features has been demonstrated in [66].

In comparison with deep learning techniques, we previously applied deep learning networks to the LABC dataset in a previous study [67], achieving a maximum accuracy of 77%. However, in this study, we attained an 80% accuracy using machine learning and wavelet radiomics. The reason for this may be attributed to the small size of our dataset, which hindered the effective training of the deep learning model.

The overfitting and generalizability challenges for small-size datasets are highly correlated with the type of machine learning algorithm. For instance, SVM is a risk minimizing algorithm and robust to overfitting for small datasets. SVM obtains a hyperplane to separate two classes by maximizing the margin between two classes.

This study demonstrated that qCT textural features could be utilized to predict the response to NAC for LABC patients, and the results indicated the effectiveness of these features in terms of sensitivity and specificity. Additionally, this study indicated the role of determined features at different levels of frequency using wavelet decomposition to improve the performance of prediction. Classifying the LABC patients that did not respond to NAC treatment is a challenge, and any changes in standard treatment can lead to complications for responder LABC patients. To this end, an equal importance weight was considered for both non-responders and responders to establish a balance between sensitivity and specificity.

The goal of this research was to develop an expert recommender system to optimize chemotherapy treatment. Physicians can use this artificial-based system to modify treatment and increase its efficiency. This system utilizes CT images and machine learning algorithms to predict whether a patient will respond to standard chemotherapy or if the regimen should be altered. The dataset size was a main limitation of our study, which can limit generalizability. Training machine learning models using a large dataset leads to better generalizability. Moreover, an external cohort validation dataset can be effective for testing the robustness of the technique and indicates the generalizability of the algorithm. Additionally, all patients in this study came from a center, and although this is useful for machine learning in terms of consistency, multi-center data improve the generalizability of algorithms by learning on different types of data, but they can be contaminated by variability linked to different practices at different sites.

The performance of a classifier can be improved by a combination of clinical features such as Nottingham grad and HER with radiomics features. Additionally, using generative models such as diffusion probabilistic methods can be efficient since they learn the distribution of data.

For breast cancer patients, mammography is used primarily for diagnosis and is not usable for therapy response prediction or the monitoring of responses. MRI is also primarily used for diagnosis. Its use for predicting and monitoring responses during chemotherapy remains in the research domain. Although NAC can also eradicate micrometastasis, with locally advanced breast cancer, NAC is often administered to downstage disease. Data from our research using different imaging modalities also indicate that local responses of gross tumor translate into the response of micrometastatic disease, as features of tumors receiving NAC can also be used as independent predictors of survival [68,69]. Hence, the proof of concept is not partially wrong.

For clinical usage and financial limitations: (a) a CT-based methodology permits the prediction (on an individual basis) of whether or not there will be a response to NAC, and it complements tests such an oncotype or mammoprint tests, which provide risk-based information on tumors linked to population data on whether chemotherapy is indicated or not. (b) A CT methodology provides a prediction of local control, and tests of this nature can also be used to predict disease-free survival and overall survival [68]. The work here focused on optimizing a methodology for the prediction of local control. (c) The methodology here offers cost-efficiency. CT-data are by default acquired in the standard work-up of patients with LABC and hence readily clinically available. Multiple scans can be rapidly obtained without the need for more expensive MRI infrastructure or more expensive radionuclide-utilizing technologies such as PET-CT, both of which have also been used for therapy response prediction.

The primary challenge of this study was the limited dataset size. Generalizability is needed for clinicians when making treatment decisions. Large and diverse datasets enhance generalizability and reduce bias, leading to more reliable and unbiased outcomes. A robust model with minimal uncertainty is typically trained on extensive and diverse datasets, resulting in more dependable predictions. With good sensitivity and specificity, models can be used, especially in scenarios where there is no clinical tool to provide comparable information.

In conclusion, a new expert system based on qCT was proposed to predict chemotherapy treatment response for patients with LABC before starting the treatment. In this method, textural features of CT images and wavelet decompositions of CT images are determined to train the learning algorithm for treatment response prediction. Using wavelet decomposition to generate features at different levels of frequency provides a comprehensive features matrix, which increase the performance of the machine learning algorithm. The results of this pilot study in terms of accuracy of prediction are promising and show that this algorithm can be used as recommender system to show NAC response prior to starting the treatment. The clinicians can use this predictive model as a recommendation system to pre-assess NAC treatment prior to starting. We found that wavelet radiomics features improve the accuracy of prediction. Additionally, feature selection plays a significant role for machine learning classifiers.

## Figures and Tables

**Table 1:** Extracted radiomics features of original image and wavelet coefficients.

Radiomics Features Type	Radiomics Features	
First Order Features:	Energy Total Energy Entropy Minimum 10th percentile 90th percentile Maximum Mean Median	Interquartile Range Range Mean Absolute Deviation (MAD) Robust Mean Absolute Deviation Root Mean Squared (RMS) Skewness Kurtosis Variance Uniformity
Shape Features:	Elongation Flatness Least Axis Length Major Axis Length Maximum 2D Diameter Column Maximum 2D Diameter Row Maximum 2D Diameter Slice	Maximum 3D Diameter Mesh Volume Minor Axis Length Sphericity Surface Area Surface Volume Ratio Voxel Volume
GLCM:	Autocorrelation Joint Average Cluster Prominence Cluster Shade Cluster Tendency Contrast Correlation Difference Average Difference Entropy Difference Variance Dissimilarity Joint Energy	Joint Entropy Homogeneity 1 Homogeneity 2 Informational Measure of Correlation (IMC) 1 Informational Measure of Correlation (IMC) 2 Inverse Difference Moment (IDM) Maximal Correlation Coefficient (MCC) Inverse Difference Moment Normalized (IDMN) Inverse Difference (ID) Inverse Difference Normalized (IDN) Inverse Variance Maximum Probability Sum Average Sum Variance Sum Entropy Sum of Squares
GLRLM:	Short Run Emphasis (SRE) Long Run Emphasis (LRE) Gray Level Non-Uniformity (GLN) Gray Level Non-Uniformity Normalized (GLNN) Run Length Non-Uniformity (RLN) Run Length Non-Uniformity Normalized (RLNN) Long Run Low Gray Level Emphasis (LRLGLE) Long Run High Gray Level Emphasis (LRHGLE)	Run Percentage (RP) Gray Level Variance (GLV) Run Variance (RV) Run Entropy (RE) Low Gray Level Run Emphasis (LGLRE) High Gray Level Run Emphasis (HGLRE) Short Run Low Gray Level Emphasis (SRLGLE) Short Run High Gray Level Emphasis (SRHGLE) Long Run Low Gray Level Emphasis (LRLGLE) Long Run High Gray Level Emphasis (LRHGLE)
GLSZM:	Small Area Emphasis (SAE) Large Area Emphasis (LAE) Gray Level Non-Uniformity (GLN) Gray Level Non-Uniformity Normalized (GLNN) Size-Zone Non-Uniformity (SZN) Size-Zone Non-Uniformity Normalized (SZNN) Zone Percentage (ZP) Gray Level Variance (GLV)	Zone Variance (ZV) Zone Entropy (ZE) Low Gray Level Zone Emphasis (LGLZE) High Gray Level Zone Emphasis (HGLZE) Small Area Low Gray Level Emphasis (SALGLE) Small Area High Gray Level Emphasis (SAHGLE) Large Area Low Gray Level Emphasis (LALGLE) Large Area High Gray Level Emphasis (LAHGLE)
GLDM:	Small Dependence Emphasis (SDE) Large Dependence Emphasis (LDE) Gray Level Non-Uniformity (GLN) Gray Level Non-Uniformity Normalized (GLNN) Dependence Non-Uniformity (DN) Dependence Non-Uniformity Normalized (DNN) Gray Level Variance (GLV)	Dependence Variance (DV) Dependence Entropy (DE) Dependence Percentage Low Gray Level Emphasis (LGLE) High Gray Level Emphasis (HGLE) Small Dependence Low Gray Level Emphasis (SDLGLE) Small Dependence High Gray Level Emphasis (SDHGLE)
NGLDM:	Coarseness Contrast Busyness Complexity Strength	

**Table 2:** Clinical the pathological and clinical characteristics of patients.

Characteristics	Responders Mean (Std)	Non-Responders Mean (Std)
**Age**	52 (11)	54 (10)
**Initial Tumor Size**	5.2 (2.5) cm	5.6 (2.7) cm
**Histology**	Percentage (Count)	
IDC	58 (70)	23 (65)
ILC	1 (1)	4 (11)
IMC	3 (3)	2 (5)
**Molecular Features**	Percentage (Count)	
ER+	42 (51)	29 (82)
PR+	37 (45)	24 (68)
^†^ HER2+	28 (34)	9 (26)
ER-/PR-/HER2-	22 (27)	4 (11)
ER+/PR+/HER2+	15 (18)	6 (17)
ER+/PR+/HER2-	22 (27)	20 (57)
ER-/PR-/HER2+	15 (18)	4 (11)
**Residual Tumor Size**	1.4 (2.4) cm	6 (5.5) cm
**Response**	Percentage (Count)	
Responding Patients	70 (82)	-
Non-responding Patients	-	30 (35)

Std = Standard Deviation, IDC = Invasive Ductal Carcinoma, ILC = Invasive Lobular Carcinoma, IMC = Invasive Metaplastic Carcinoma, ER = estrogen, PR= progesterone. †: Statistically significant.

**Table 3:** Performance of the outcome prediction for all three models using hold-out data splitting.

	Classifier	FST	# Features	Spec		Sens		Acc		B-Acc	
				Mean	Max	Mean	Max	Mean	Max	Mean	Max
				(%)		(%)		(%)		(%)	
Model 1:	KNN	mRMR	Top-5	77	86	51	60	72	77	62	76
Model 2:	KNN	mRMR	Top-10	78	88	52	58	74	78	64	73
Model 3:	KNN	mRMR	Top-5	80	90	56	63	77	79	68	77

Model 1: Only Image Features, Model 2: Only Wavelet Features, Model 3: Image and Wavelet Features, B-Acc: Balanced Accuracy, Acc: Accuracy, Sens: Sensitivity, Spec: Specificity, FST: Feature Selection Technique, # Features: The Number of Selected Features which led to maximum accuracy.

**Table 4:** Performance of the outcome prediction for all three models using LOPO splitting.

	Classifier	FST	# Features	Spec (%)	Sens (%)	Acc (%)	B-Acc (%)
Model 1:	KNN	mRMR	Top-5	69	58	68	66
Model 2:	KNN	mRMR	Top-10	71	61	71	70
Model 3:	KNN	mRMR	Top-5	76	62	75	72

Model 1: Only Image Features, Model 2: Only Wavelet Features, Model 3: Image and Wavelet Features, B-Acc: Balanced Accuracy, Acc: Accuracy, Sens: Sensitivity, Spec: Specificity, FST: Feature Selection Technique, # Features: The Number of Selected Features which led to maximum accuracy.

**Table 5:** Performance of different feature selection techniques for LOPO cross-validation using original image and wavelet features.

Technique/Metric	Specificity	Sensitivity	Accuracy	B-Accuracy
mRMR	76	62	75	72
Relief	78	36	64	57
PFS	81	35	67	56
Rref	86	30	69	57
QR	82	39	70	60
NMFFS	71	35	60	54

B-Accuracy: Balanced Accuracy.

## Data Availability

Data collected and analyzed in this study are available from the Sunnybrook Research Institute Research Ethics Board approved study ‘Pilot Investigation of Ultrasound Imaging and Spectroscopy and Ultrasound Imaging of Vascular Blood Flow as Early Indicators of Locally Advanced Breast Cancer Response to Neoadjuvant Treatment’. Since this is patient data, the authors are legally bound to keep it confidential. Data can be made available upon request and review by Institutional Review Board (IRB). Data requests may be sent to Dr. Kullervo Hynynen, Vice-president, Research & Innovation, Sunnybrook Research Institute (khynynen@sri.utoronto.ca).

## Supplementary Materials

The following supporting information can be downloaded at: https://www.mdpi.com/article/10.3390/tomography11030033/s1, Section S1 [70,71,72], Section S2 [47,48,49,51,59,73,74,75,76,77,78,79,80,81,82,83,84,85,86,87,88,89,90,91], Figure S1: This figure illustrates Level-1 and Level-2 decompositions by wavelet transform. (a) Where A, H, V and D represent approximation, horizontal, vertical and diagonal coefficients. (b) Level-2 discrete wavelet transform decomposition of image into eight sub-band, Figure S2: The frequency of selected features in each iteration for all features (original image and wavelet coefficients), Figure S3: The frequency of selected features in each iteration for original image radiomic features, Figure S4: Frequency of selected features for wavelet coefficient; (a) HHH, (b) HHL, (c) HLH, (d) HLL, Figure S5: Frequency of selected features for wavelet coefficient; (a) LHH, (b) LHL, (c) LLH, (d) LLL, Table S1: Performance of the outcome prediction models for hold-out cross validation using original image and wavelet features, Table S2: Performance of the outcome prediction models for LOPO cross validation using only original image, Table S3: Performance of the outcome prediction models for LOPO cross validation using only wavelet features, Table S4: Performance of the outcome prediction models for LOPO cross validation using original image and wavelet features, Table S5: Two-side t-test.
