# Supplementary material for: Prediction of Chemotherapy Response in Locally Advanced Breast Cancer Patients at Pre-Treatment Using CT Textural Features and Machine Learning: Comparison of Feature Selection Methods
Source: Tomography. 2025 Mar 13;11(3):33. doi: 10.3390/tomography11030033 (PMC11946754; doi:10.3390/tomography11030033)
Supplement: Supplementary file 1 [file tomography-11-00033-s001.zip › tomography-3290287-supplementary.pdf]

Supplementary Materials

S1 Feature extraction

Wavelet feature extraction

2-D Fast Fourier Transform (FFT) is frequently used to represent image in frequencies domain. Resolving in frequency domain and losing time information are the major drawbacks with Fourier Transform. Short Time Fourier Transform (STFT) is proposed as a alternative for time frequency analysis. In STFT, a window with specific shape and size is applied to the image/signal to compute Fourier Transform. In time-frequency analysis, getting complete information in both the time and frequency domains is limited by uncertainty principle limit. To this end, Wavelet transform can be applied to preserve information in both the time and frequency [1–3].

The basic idea in wavelet analysis is to use a mother wavelet (haar, dmey, sym and bior) to generate a family of scaled and translated versions of the image. Then, type of wavelet and level of decomposition are two main parameters should be determined for radiomic feature extraction using wavelet transform. In Figure S1 the frequency decomposition using wavelet is shown.

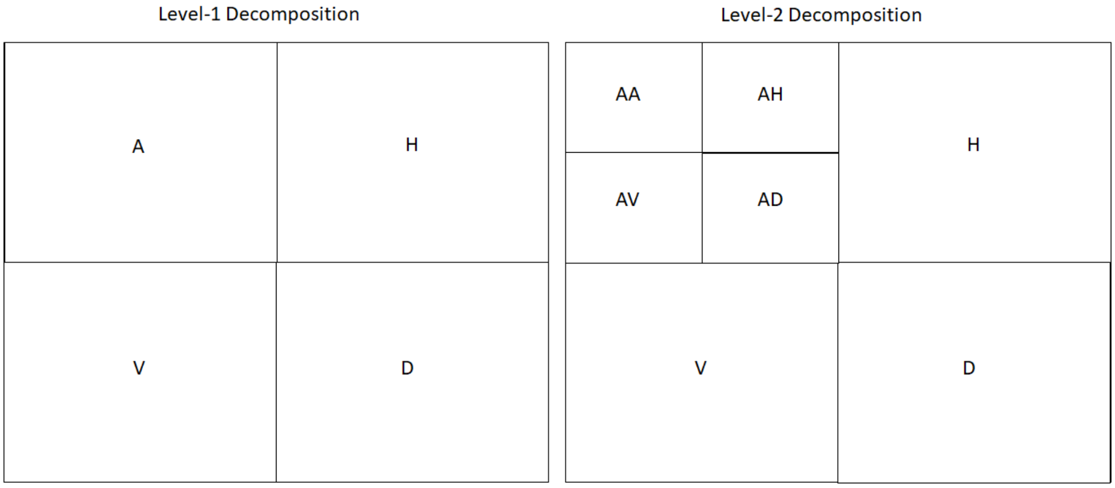

(a)

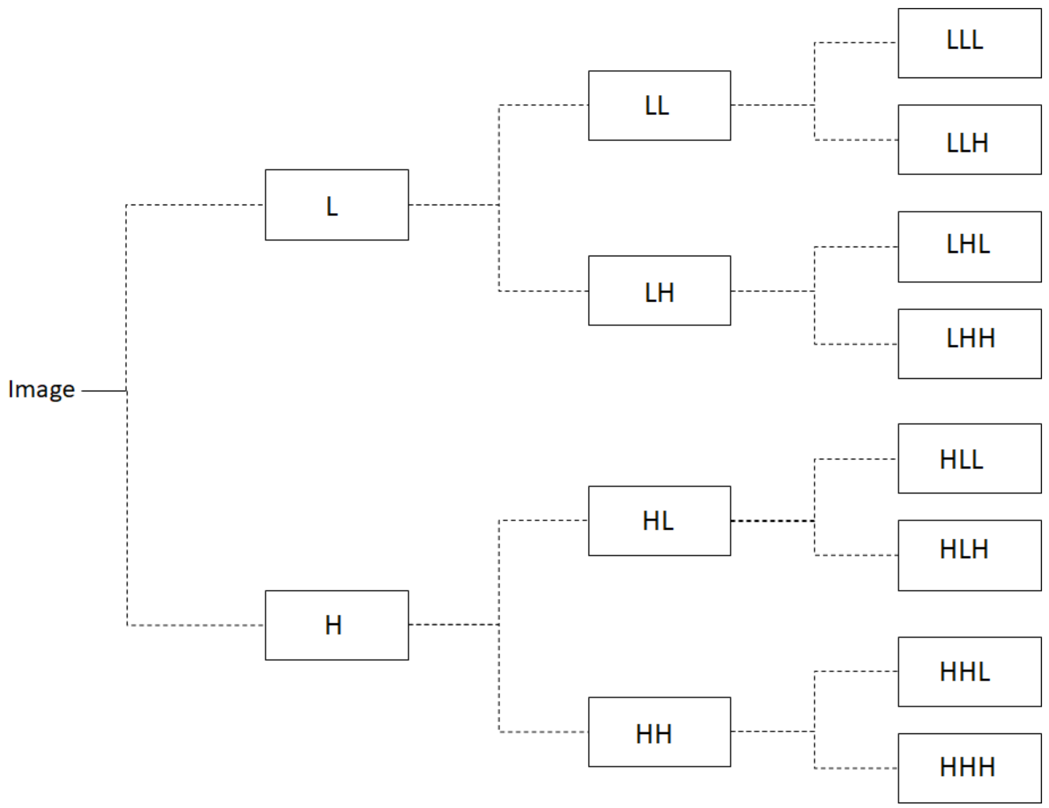

(b)

Figure S1. This figure illustrates Level-1 and Level-2 decompositions by wavelet transform. (a) Where A, H, V and D represent approximation, horizontal, vertical and diagonal coefficients. (b) Level-2 discrete wavelet transform decomposition of image into eight sub-band.

Where L and H in Figure S1 (b) are low-pass and high-pass frequency filters, respectively. Likewise, LL, LH, HL and HH are approximation (A), horizontal (H), vertical (V) and diagonal (D) coefficients, respectively.

S2 Feature selection

Feature selection techniques based on label information is categorized to supervised, unsupervised and semi-supervised techniques, and based on strategy is classified to Filter-based, Wrapper-based and embedded-based strategies.

In the context of supervise feature selection, important features are selected based on the interaction between features and labels [4]. In semi-supervised, the labels of all samples are not available and information about unlabeled data are extracted based on labeled data [5]. For no label information available scenario which is called unsupervised, and so feature selection can be applied based on the relevancy among features are measured to select important features [6].

In the context of search strategy, filter strategy finds important feature without connection between classifier and dataset using Laplacian score [7], information theory-based techniques such as mutual information [8–11], and mRMR [12], statistic tests and matrix factorization-based technique [13,14] are common methods that are applied for feature selection. In this strategy, the relevancy of features and labels is evaluated, and irrelevant features are removed [15–19]. In Wrapper strategy, dataset and classifier are coupled to find most important features based on the output of classifier. sequential forward feature selection, sequential backward feature selection and floating search [20] and recursive SVM [21] are conventional wrapper-based feature selection techniques. Embedding the features selection in training process is known as embedded strategy for feature selection. For instance, information gain or Gini-index are employed as criteria for splitting features in decision tree.

mRMR

mrmr is a feature selection which is constructed based on maximum relevancy and minimum redundancy concept. Maximum relevancy and minimum redundancy are defined based on mutual information between label and feature and two features, respectively [12]. As a result, following objective function is considered for mrmr;

$$\max \frac{1}{|S|}\sum_{a_i \in S} I(a_i; c) - \frac{1}{|S|}\sum_{a_j, a_i \in S} I\left(a_i; a_j\right) \quad (1)$$

Where S, I, a_i, a_j and c are feature set, mutual information, feature i, feature j and class label, respectively. For implementation, in matlab, use fcmrmr command.

QR feature selection

QR is an unsupervised filter-based feature selection, which is working based on subspace learning. In this technique, column space of matrix is extracted and a permutation matrix is obtained which shows the relation between column space and features in original space. This is unique characteristic of QR, which allows applying for feature selection. Other matrix factorization techniques including nonnegative matrix factorization (NMF) and singular value decomposition cannot be directly applied feature selection. Although NMF is recently drawn attention for feature selection, this technique cannot be directly used for feature selection and requires orthogonality constraint. However, it has been shown by example $\begin{bmatrix} 1/\sqrt{2} & 0 & 1/2 \\ 0 & 1 & 0 \end{bmatrix}$ that orthogonality condition is not solely sufficient for feature selection [22]. For implementation, in matlab, [Q,R,P] = qr(A).

Nonnegative matrix factorization feature selection (NMFFS) [23]: NMFFS is an unsupervised feature selection based on filter strategy. In this technique, data is decomposed into two nonnegative matrices including feature weight matrix and representation matrix. We can have feature weight matrix as indictor matrix by imposing orthogonality constraint on feature weight matrix. Consequently, Euclidian norm of each in feature weight matrix shows the importance of each feature of data. (Pseudo code can be obtained in [23] Algorithm 1)

Relief

Relief algorithm is the one of the classical feature selection techniques to obtain important features. The Relief algorithm, m samples of data are randomly selected, and for each

sample the nearest neighbors (NN) of same class and opposite class are obtained [24]. In this technique, the importance of feature is assessed based on the quality of differentiation between same class and opposite class. Consequently, a feature can be considered as an important feature if it can distinguish two close samples that belong to different classes [25]. For implementation, in matlab, use Relieff command.

Perturbation-based technique

The number of features can be greater than samples size in some datasets. Most of these types of datasets are ill-posed condition which means that the matrix of data has big condition number. The role of each feature for increasing and decreasing condition number can be evaluated as a metric to obtain most important features. To this end, perturbation theory can be leveraged to obtain important features based on variation in condition number of data-matrix [26]. In this technique, redundant features, which are main reason of high condition number, can be detected using a comparison between perturbed data and unperturbed data. This method solves least square equation $Ax=b$ and $(A+E)\dot{x}=b$ using the normal equation technique to calculate x and $\dot{x}$ and $\Delta x=x-\dot{x}$. Where A, E and b are original feature matrix, perturbation matrix and target, respectively. solutions are $x=(A^TA)^{-1}A^Tb$ and $\dot{x}=((A+E)^T(A+E))^{-1}(A+E)^Tb$, where T is the transpose. Likewise, Δx is considered as a metric to measure the correlation of each feature with other features. In next step, K-means clustering algorithm is leveraged to cluster the features based on Δx similarity. In final step, features are ranked in each cluster based on entropy metric and first feature in each cluster is consider as selected feature. (matlab code can be found in http://github.com/majid1292/DRPT)

Reduced row echelon form (Rref) [27]: Rref is filter-based supervised feature selection technique. Rref sorts all features based on information gain and apply reduced row echelon form to extract all independent features. For implementation, Rref algorithm

just needs to sort features based on information gain and apply [R,p] = rref(A) matlab command.

Classifier Hyper parameters:

The number of neighbors is the key hyper-parameter of KNN which test data is predicted based on class majority of neighbors. Parameter “C” and “gamma” are two hyper-parameters of SVM. Parameter “C” provides a trade-off between non-separable samples and complexity algorithm by restricting the Lagrange multipliers and parameter “gamma” is the radius of RBF kernel. The maximum depth of tree in DT is main hyper parameter which is directly related to complexity of algorithm and the probability of overfitting.

Results

Table S1. Performance of the outcome prediction models for hold-out cross validation using original image and wavelet features.

	Selected Features	Specificity Mean %	Specificity Max %	Sensitivity Mean %	Sensitivity Max %	Accuracy Mean %	Accuracy Max %	B-Accuracy Mean %	B-Accuracy Max %
DT-mRMR	Top-5	38	90	67	63	60	76	53	77
	Top-10	57	75	55	75	57	64	56	75
	Top-15	62	70	40	87	56	65	51	66
SVM-mRMR	Top-5	58	75	56	65	57	68	57	70
	Top-10	72	88	46	55	53	65	59	71
	Top-15	77	88	45	50	54	61	62	72
KNN-mRMR	Top-5	**80**	90	**56**	63	**77**	79	**68**	77
	Top-10	77	85	33	50	65	75	55	68
	Top-15	72	80	45	75	64	79	59	78
DT-pert*	Top-5	48	100	60	75	56	68	54	75
	Top-10	38	75	53	65	63	75	44	59

	Top-15	43	75	46	70	45	65	45	60
SVM-pert*	Top-5	77	100	27	55	41	54	52	64
	Top-10	70	100	33	50	44	57	52	63
	Top-15	63	100	27	35	36	47	45	53
KNN-pert	Top-5	40	75	53	74	50	65	44	60
	Top-10	68	88	44	50	51	61	56	69
	Top-15	43	63	38	45	40	43	41	55
DT-Relief	Top-5	58	80	38	63	52	71	48	65
	Top-10	65	70	27	63	54	60	46	67
	Top-15	68	85	33	63	58	71	51	74
SVM-Relief	Top-5	62	100	50	80	53	71	56	65
	Top-10	58	75	41	65	45	65	50	64
	Top-15	70	100	36	45	43	61	48	68
KNN-Relief	Top-5	84	95	20	38	66	71	52	66
	Top-10	73	80	23	38	60	68	48	59
	Top-15	73	80	28	64	60	68	51	72
DT-QR	Top-5	59	90	40	87	54	68	50	60
	Top-10	53	90	53	100	53	68	53	60
	Top-15	56	80	50	100	54	64	53	64
SVM-QR	Top-5	64	100	51	82	55	74	58	69
	Top-10	60	76	43	67	47	66	53	64
	Top-15	72	100	38	47	45	63	51	71
KNN-QR	Top-5	78	100	50	63	72	79	64	63
	Top-10	77	85	33	50	65	75	55	68
	Top-15	76	100	33	63	64	79	54	64

B-Accuracy= Balanced Accuracy, pert*=Perturbation, DT=Decision Tree, SVM=Support vector machine

Table S2. Performance of the outcome prediction models for LOPO cross validation using only original image.

	Specificity	Sensitivity	Accuracy	Balanced-Ac
MRMR				
KNN-5	**69**	**58**	**68**	**66**
SVM-5	96	4	68	48
DT-5	78	40	67	59
KNN-10	71	29	58	50
SVM-10	61	40	55	50
DT-10	70	40	61	55
KNN-15	67	31	56	49
SVM-15	57	37	51	47
DT-15	68	34	58	51
Perturbation				
KNN-5	20	73	57	47
SVM-5	26	57	48	42
DT-5	40	71	62	55
KNN-10	23	75	61	51
SVM-10	29	59	48	45
DT-10	43	74	65	58
KNN-15	70	19	54	42
SVM-15	55	23	44	36
DT-15	67	38	59	51
Relief				
KNN-5	70	31	58	50
SVM-5	48	51	49	49
DT-5	61	17	48	39
KNN-10	70	29	57	49
SVM-10	49	51	50	50
DT-10	63	29	53	46
KNN-15	70	26	56	48
SVM-15	74	29	61	51
DT-15	63	26	52	45
Rref				
KNN-5	79	31	65	55

SVM-5	83	20	64	51
DT-5	68	29	56	48
KNN-10	77	14	58	46
SVM-10	67	26	55	46
DT-10	73	43	64	58
KNN-15	62	34	54	48
SVM-15	62	37	55	50
DT-15	73	34	62	54
QR				
KNN-5	73	17	56	45
SVM-5	99	0	69	49
DT-5	77	34	64	56
KNN-10	73	17	56	45
SVM-10	99	0	69	49
DT-10	70	23	56	46
KNN-15	73	17	56	45
SVM-15	99	0	69	49
DT-15	73	37	62	55

B-Accuracy= Balanced Accuracy, pert*=Perturbation, DT=Decision Tree, SVM=Support vector machine

Table S3. Performance of the outcome prediction models for LOPO cross validation using only wavelet features.

	Specificity	Sensitivity	Accuracy	Balanced-Ac
**MRMR**				
KNN-5	79	29	64	54
SVM-5	76	26	61	51
DT-5	73	37	62	55
KNN-10	82	26	65	54
SVM-10	77	31	63	54
DT-10	74	29	61	51
KNN-15	83	26	66	54
SVM-15	73	31	61	52
DT-15	74	20	58	47
**Perturbation**				

KNN-5	79	14	60	47
SVM-5	82	14	62	48
DT-5	77	17	59	47
KNN-10	77	16	59	46
SVM-10	79	17	60	44
DT-10	76	18	58	45
KNN-15	74	13	55	42
SVM-15	75	12	54	41
DT-15	74	16	56	43
**Relief**				
KNN-5	82	20	63	51
SVM-5	100	0	70	50
DT-5	70	26	56	48
KNN-10	80	14	61	47
SVM-10	100	0	70	50
DT-10	74	51	68	63
KNN-15	79	9	58	44
SVM-15	100	0	70	50
DT-15	73	37	62	55
**Rref**				
KNN-5	82	23	64	52
SVM-5	66	26	54	46
DT-5	72	34	61	53
KNN-10	87	26	68	56
SVM-10	61	40	55	50
DT-10	72	37	62	55
KNN-15	76	23	60	49
SVM-15	59	9	44	34
DT-15	67	43	60	55
**QR**				
KNN-5	84	34	69	59
SVM-5	99	0	69	49
DT-5	70	29	57	49
KNN-10	82	31	67	57
SVM-10	99	0	69	49
DT-10	**61**	**71**	**68**	**66**

KNN-15	82	31	67	57
SVM-15	99	0	69	49
DT-15	73	37	62	55

B-Accuracy= Balanced Accuracy, pert*=Perturbation, DT=Decision Tree, SVM=Support vector machine

Table S4. Performance of the outcome prediction models for LOPO cross validation using original image and wavelet features.

	Specificity	Sensitivity	Accuracy	Balanced-Ac
**MRMR**				
KNN-5	**76**	**62**	**75**	**69**
SVM-5	80	13	59	47
DT-5	69	55	65	63
KNN-10	72	57	60	66
SVM-10	78	14	57	46
DT-10	67	53	58	60
KNN-15	69	53	57	51
SVM-15	76	12	54	44
DT-15	65	17	54	55
**Perturbation**				
KNN-5	81	19	64	51
SVM-5	80	18	63	49
DT-5	78	20	62	49
KNN-10	80	19	63	50
SVM-10	80	20	62	46
DT-10	81	19	61	51
KNN-15	77	17	59	47
SVM-15	77	16	57	46
DT-15	72	22	58	47
**Relief**				
KNN-5	84	23	66	54
SVM-5	95	8	71	52
DT-5	74	29	59	51
KNN-10	78	22	64	50
SVM-10	100	0	70	50
DT-10	75	53	70	65

KNN-15	80	12	59	46
SVM-15	100	0	70	50
DT-15	78	36	64	57
**Rref**				
KNN-5	83	25	65	54
SVM-5	64	31	56	48
DT-5	74	37	63	55
KNN-10	86	30	69	57
SVM-10	63	41	56	51
DT-10	73	39	63	56
KNN-15	75	29	62	51
SVM-15	58	18	48	38
DT-15	68	46	62	57
**QR**				
KNN-5	82	39	70	60
SVM-5	100	0	70	50
DT-5	70	29	57	49
KNN-10	80	39	69	59
SVM-10	100	0	70	50
DT-10	72	62	69	67
KNN-15	83	36	70	60
SVM-15	100	0	70	50
DT-15	75	39	64	57

B-Accuracy= Balanced Accuracy, pert*=Perturbation, DT=Decision Tree, SVM=Support vector machine

The histogram of selected features based on frequency of selection in each iteration for 851 features is shown in Figure E4. The histogram of selected features for image and wavelet coefficients are shown individually in Figures E5, E6 and E7, respectively.

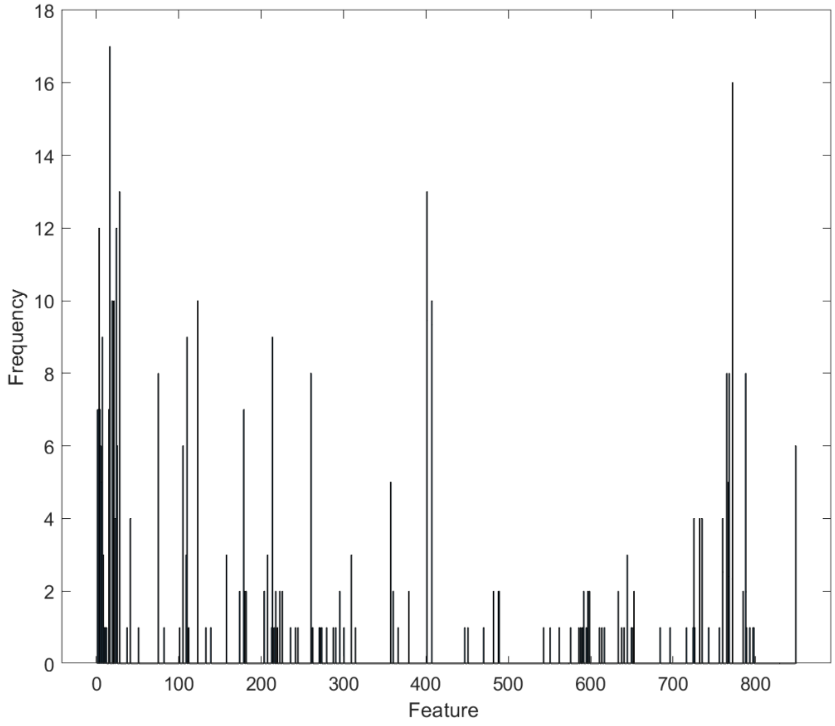

Figure S2. The frequency of selected features in each iteration for all features (original image and wavelet coefficients)

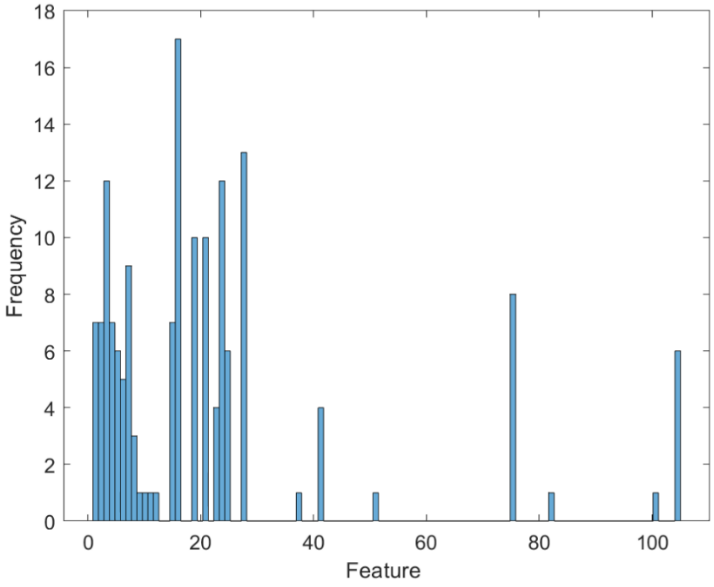

Figure S3. The frequency of selected features in each iteration for original image radiomic features.

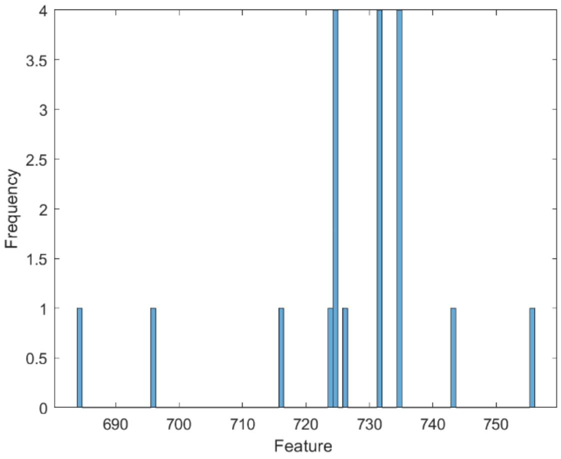

(a)

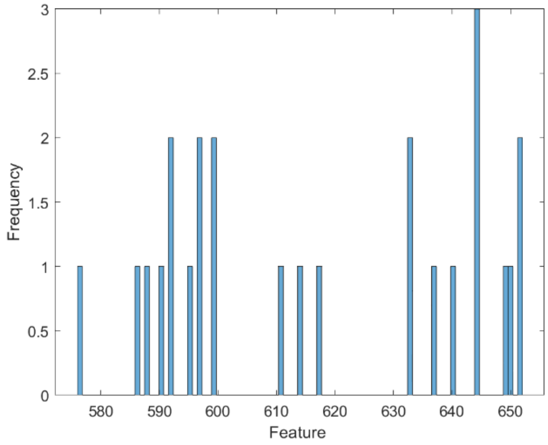

(b)

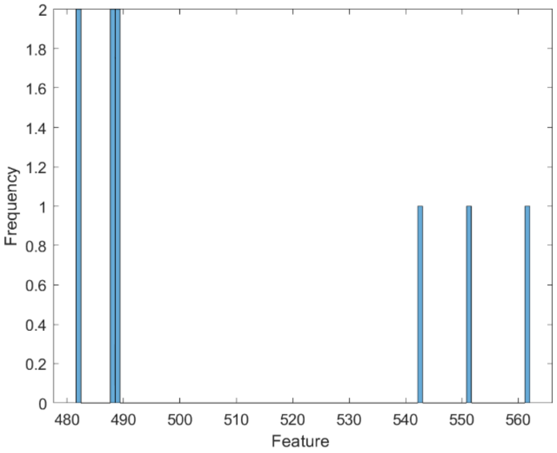

(c)

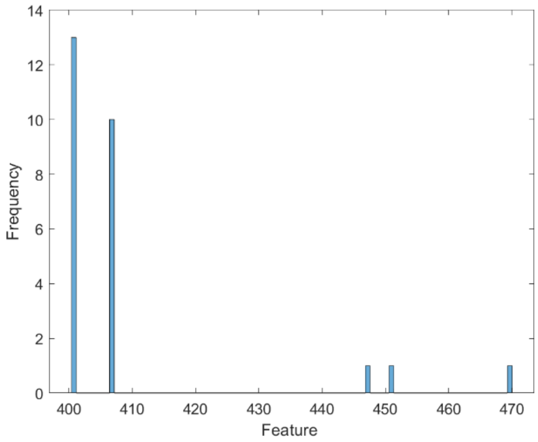

(d)

Figure S4. Frequency of selected features for wavelet coefficient; (a) HHH, (b) HHL, (c) HLH, (d) HLL.

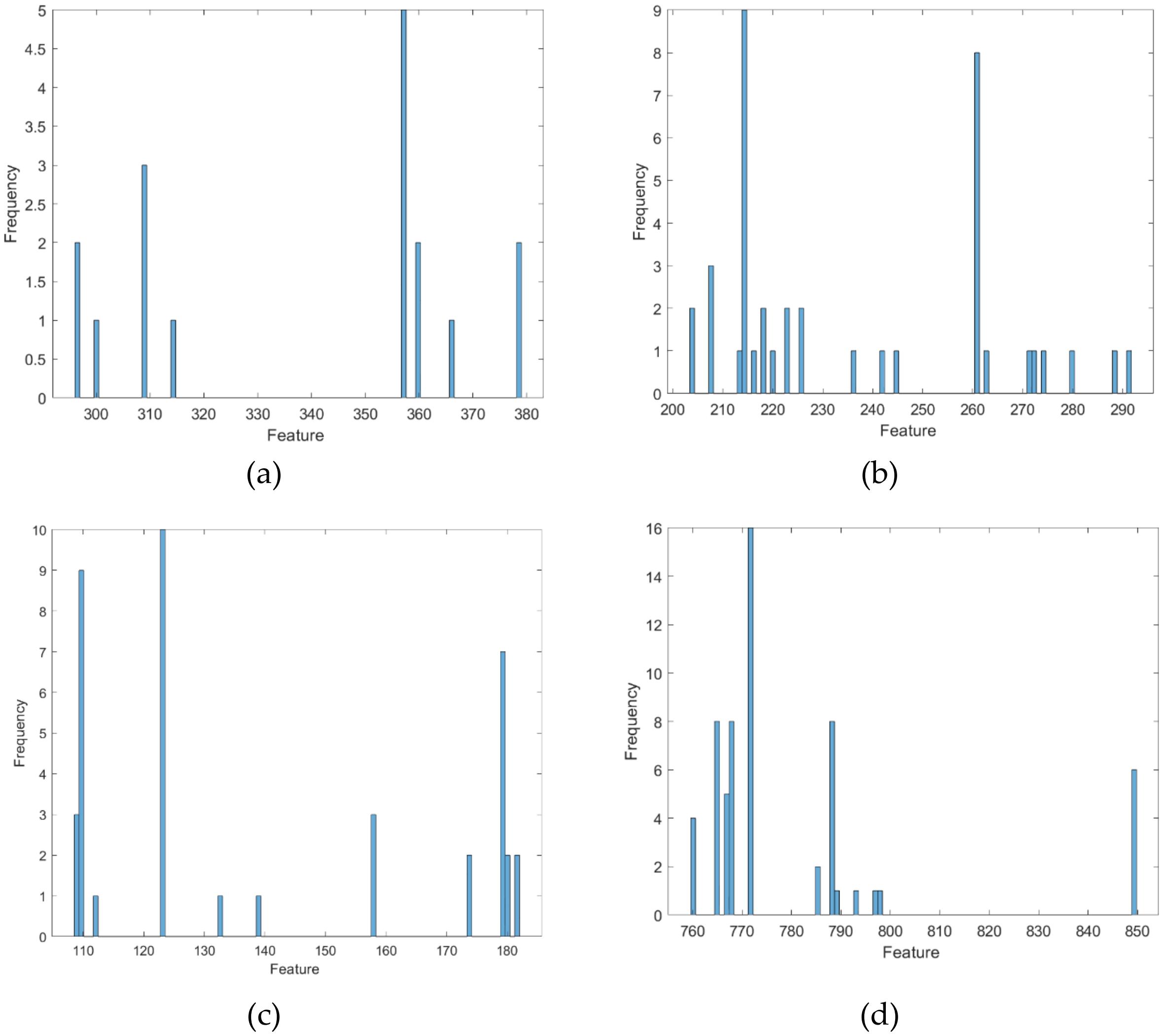

Figure S5. Frequency of selected features for wavelet coefficient; (a) LHH, (b) LHL, (c) LLH, (d) LLL.

t-test was applied to compare non-responder and responder cohorts and *p*-value of selected features is shown in Table E5.

Table S5. Two-side t-test

Features	p-value
Wavelet-LLH-GLDM- Dependence Entropy	**0.04**
Wavelet-HHL-GLCM-Difference variance	0.34
Wavelet-HLL-GLCM-Cluster Shade	0.32
Wavelet-LLH-GLDM- Small Dependence Emphasis	0.42

Wavelet-HHH-GLSZM- Small Area High Gray Level Emphasis	0.51
Wavelet-LLL-GLCM-Idmn	0.51
Original-First Order-Median	0.50
Wavelet-LLL-First Order- Maximum	0.52
Original-First Order- Root Mean Square	0.48
Wavelet-HLL-First Order- Skewness	0.47
Wavelet-HHH-GLSZM-Size Zone Non-Uniformity	0.43
Wavelet-HHL-First Order- Mean Absolute Deviation	0.43
Wavelet-LLL-First Order- 90 Percentile	0.41
Original-GLCM-Gray level variance	0.44
Wavelet-LLH-GLSZM-Zone Entropy	0.39
Wavelet-HHL-GLCM- Joint Entropy	0.41
Wavelet-LLH-First Order-Energy	0.42
Original-First Order- Root Mean Square	0.43
Wavelet-LHL-GLSZM-Gray Level Variance	0.45
Wavelet-HHH-GLCM- Joint Entropy	0.42

References

1. Mark A. Pinsky. Introduction to Fourier analysis and wavelets, volume 102. American Mathematical Soc., 2002.
2. Brunton, S. L. & Kutz, J. N. Data-Driven Science and Engineering: Machine Learning, Dynamical Systems and Control (Cambridge Univ. Press, 2019).
3. Nason, G.P., Silverman, B.W. (1995). The Stationary Wavelet Transform and some Statistical Applications. In: Antoniadis, A., Oppenheim, G. (eds) Wavelets and Statistics. Lecture Notes in Statistics, vol 103. Springer, New York, NY. https://doi.org/10.1007/978-1-4612-2544-7_17
4. L. Wolf, A. Shashua, “Feature selection for unsupervised and supervised inference: the emergence of sparsity in a weight based approach”, J. Mach. Learn. Res. 6 (2005) 1855–1887.
5. Z. Zhao, H. Liu, “Semi-supervised feature selection via spectral analysis”, in: SDM, SIAM, 2007, pp. 641–646.
6. D. Cai, C. Zhang, X. He, “Unsupervised feature selection for multi-cluster data”, in: Proceedings of the 16th ACM SIGKDD international conference on Knowledge discovery and data mining, AC „ 2010, pp. 333–342.
7. X. He ,” Laplacian score for feature selection”, in: Advances in neural information processing systems, 2005, pp. 507–514
8. R. Battiti , “Using mutual information for selecting features in supervised neural net learning”, IEEE Trans Neural Netw. 5 (4) (1994) 537–550 .
9. C. Lazar , “A survey on filter techniques for feature selection in gene expression microarray analysis, IEEE/ACM Trans. Comput. Biol. Bioinform. 9 (4) (2012) 1106–1119 .
10. G. Forman , An extensive empirical study of feature selection metrics for text clas- sification, J. Mach. Learn. Res. 3 (2003) 1289–1306 .
11. N. Kwak , C. Choi , Input feature selection for classification problems, IEEE Trans. Neural Netw. 13 (2002) 143–159 .
12. H. Peng, F. Long, and C. Ding, “Feature selection based on mutual information criteria of max-dependency, max-relevance, and min-redundancy,” IEEE Trans. Pattern Anal. Mach. Intell., vol. 27, no. 8, pp. 1226–1238, Aug. 2005.

13. L. Elden, Matrix methods in data mining and pattern recgonition, The Society for Industrial and Applied Mathematics, 2007.
14. N. Halko, P.-G. Martinsson, J. A. Tropp, Finding structure with randomness: probabilistic algorithms for constructing approximate matrix decompositions, SIAM review 53 (2) (2011) 217–288.
15. R. Kohavi ,” Wrappers for feature subset selection”, Artif. Intell. 97 (1997) 273–324 .
16. I. Guyon , “An introduction to variable and feature selection”, J. Mach. Learn. Res. 3 (2003) 1157–1182 .
17. L. P. , “Selection of relevant features in machine learning”, AAAI fall symp relevance, 1994 .
18. A. Blum ,” Selection of relevant features and examples in machine learning”, Artif. Intell. 97 (1997) 245–270 .
19. G. John , “Irrelevant features and the subset selection problem”, in: Proc. 11th int. conf. mach. learn., 1994, pp. 121–129
20. P. Pudil, J. Novovicova, and J. Kittler, “Floating Search Methods in Feature Selection,” Pattern Recognition Letters, vol. 15, no. 11, pp. 1119-1125, 1994.
21. Zhang X, et al. Recursive SVM feature selection and sample classification for mass-spectrometry and microarray data BMC Bioinformatics, 2006, vol. 7 pg. 197
22. Saberi-Movahed, Farid, Mahdi Eftekhari, and Mohammad Mohtashami. "Supervised feature selection by constituting a basis for the original space of features and matrix factorization." International Journal of Machine Learning and Cybernetics 11 (2020): 1405-1421.
23. Moslemi, Amir. "A tutorial-based survey on feature selection: Recent advancements on feature selection." Engineering Applications of Artificial Intelligence 126 (2023): 107136.
24. K.Kira,L.Rendell,”A practical approach to feature selection”, in:Proceedingsof the International Conference on Machine Learning,1992, pp. 249–256.
25. M. Robnik-Sikonja,I.Kononenko,”Theoretical and empirical analysis ofReliefF and RReliefF”,Mach.Learn.53(1–2) (2003)23–69.
26. J.R. Anaraki, H. Usefi.A feature selection based on perturbation theory. Expert Systems with Applications, 127 (2019), pp. 1-8
27. Ebrahimpour, M.K., Zare, M., Eftekharia, M., Aghamollaei, Gh, 2017. Occam’s razor in dimension reduction: using reduced row Echelon form for finding linear independent features in high dimensional microarray datasets. Eng. Appl. Artif. Intell. 62, 214–221.
